# Realizing Target Detection in SAR Images Based on Multiscale Superpixel Fusion

**DOI:** 10.3390/s21051643

**Published:** 2021-02-26

**Authors:** Ming Liu, Shichao Chen, Fugang Lu, Mengdao Xing, Jingbiao Wei

**Affiliations:** 1 Key Laboratory of Modern Teaching Technology, Ministry of Education, Xi’an 710062, China; mliu@snnu.edu.cn; 2 School of Computer Science, Shaanxi Normal University, Xi’an 710119, China; 3 No. 203 Research Institute of China Ordnance Industries, Xi’an 710065, China; lufugang203@163.com; 4 National Laboratory of Radar Signal Processing, Xidian University, Xi’an 710071, China; xmd@xidian.edu.cn; 5 Army Aviation Research Institute, Beijing 101121, China; 15911081501@163.com

**Keywords:** synthetic aperture radar (SAR) images, target detection, superpixel segmentation, fusion

## Abstract

For target detection in complex scenes of synthetic aperture radar (SAR) images, the false alarms in the land areas are hard to eliminate, especially for the ones near the coastline. Focusing on the problem, an algorithm based on the fusion of multiscale superpixel segmentations is proposed in this paper. Firstly, the SAR images are partitioned by using different scales of superpixel segmentation. For the superpixels in each scale, the land-sea segmentation is achieved by judging their statistical properties. Then, the land-sea segmentation results obtained in each scale are combined with the result of the constant false alarm rate (CFAR) detector to eliminate the false alarms located on the land areas of the SAR image. In the end, to enhance the robustness of the proposed algorithm, the detection results obtained in different scales are fused together to realize the final target detection. Experimental results on real SAR images have verified the effectiveness of the proposed algorithm.

## 1. Introduction

Synthetic aperture radar (SAR) is different from optical sensors, and is capable of penetrating rain, snow, cloud, and fog, providing high-resolution images under severe weather conditions [1,2,3]. As a result, SAR has been widely exploited in various application fields [4,5,6]. Thanks to a large number of collected SAR images, automatic target recognition (ATR) of SAR images have attracted increasing popularity in recent years. SAR ATR provides the basis of the reconnaissance of interested regions or the precise strike of threatening targets for both civil and military applications [7,8,9]. A commonly used scheme of SAR ATR proposed by the Lincoln laboratory mainly consists of three consecutive stages, which are detection, discrimination, and classification, respectively [10,11]. Many effective algorithms utilizing various advanced models have been proposed for classification, achieving overwhelming performance [12,13,14]. However, problems still exist for the first two stages before classification. This paper focuses on the detection stage, which aims to eliminate the false alarms in the images and reduce the pressure of the following stages of SAR ATR.

Due to the advantages of low-cost computation and adaptive threshold determination, the constant false alarm rate (CFAR) based methods have been the most popularly used for target detection. Focusing on target detection under different backgrounds, various CFAR-based algorithms have been proposed, such as the two-parameter CFAR detector [15], the cell-averaging CFAR (CA-CFAR) detector [16], and the smallest of CFAR (SO-CFAR) detector [17]. The two-parameter CFAR detector is suitable for simple scenes with high signal-to-clutter ratio (SCR), but its performance will degrade dramatically when the image scene is complex. Satisfying detection performance can be achieved by the CA-CFAR detector when the background clutter is homogeneous. The SO-CFAR detector leads to an increased false alarm rate, since its threshold is determined by choosing the smallest mean value of the divided background windows. Plenty of disturbances will exceed the threshold. In other words, different CFAR detectors are suitable for different image scenes. However, for complex scenes, the performance of all these detectors will degrade.

Essentially, the key point of the CFAR detection is the precise modeling of the background clutters. Plenty of advanced statistical models have been proposed, such as the generalized Gamma distribution [18], the K distribution [19], and the G0 distribution [20]. Better descriptions lead to better detection performances. The reason why the CFAR detector cannot perform well for all of the scenes is that it is very difficult to achieve accurate statistical modeling of the background clutters, especially for complex backgrounds, such as the images covered by both sea and land. The distribution of mixed textures is hard to describe precisely. Moreover, since only the intensity difference is taken into account under the CFAR detection, it is difficult to distinguish the manmade clutters, such as the buildings from the interested targets. Moreover, for complex image scenes with both land and sea, disturbances near the coastline, are hard to exclude.

Focusing on this problem, we try to fuse the land-sea segmentation into the traditional CFAR detection. In recent days, the superpixel technology has been widely used in image processing, including optical images and SAR images. Moreover, superpixel-based algorithms have achieved satisfying results [21,22,23,24]. Theoretically, any target in SAR images can be described by one superpixel or some connected superpixels [21,22,23]. Superpixels can produce an over-segmentation of an image, realizing clustering that contains similar pixels. Superpixel segmentation is capable of reflecting the boundaries and local features of images [21]. We can use superpixel segmentation to obtain a more accurate separation between land and sea in the image. As a result, we can remove the false alarms in the land areas from the CFAR detection results, especially for the ones near the coastline.

The main idea of the proposed algorithm is to utilize multiscale superpixel segmentation to better describe the local structures and properties of the complex images. In the proposed algorithm, we obtain the coarse detection result by using the traditional CFAR detector firstly. Then, the land-sea segmentation is achieved based on the statistical properties of the superpixels by using the Kullback–Leibler (KL) divergence [25] and the Kolmogorov–Smirnov (KS) distance [26]. Thirdly, the land-sea segmentations are combined with the coarse detection result to realize significant reduction of the false alarms. In the end, multiscale detection results are fused together to better enhance the robustness and accuracy of the proposed target detection algorithm.

The main contributions of this paper are summarized as follows:

1. Land-sea segmentation is realized by using the statistical property of the superpixel, which results in a more accurate separation between land and sea.

2. Superpixel segmentations of the SAR images are obtained in multiple scales. Different information can be obtained from different scales of the superpixel segmentations, which provides more information than just using one scale.

3. The detection results obtained in different scales are fused together to get the final detection result, which leads to satisfying target detection with stronger robustness and higher accuracy.

## 2. The Proposed Target Detection Algorithm

**Figure 1:**
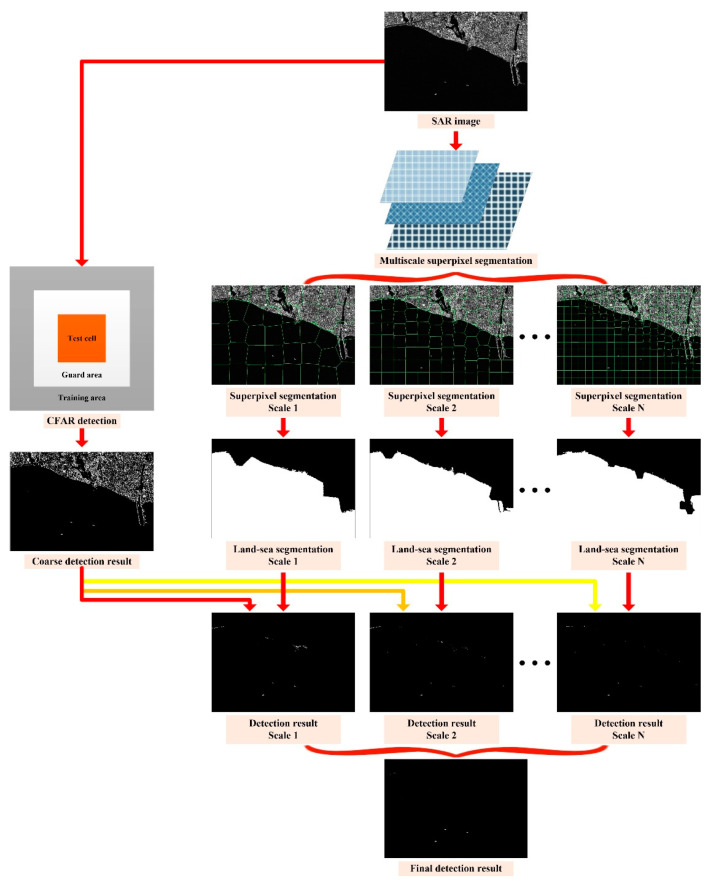
The flowchart of the proposed synthetic aperture radar (SAR) target detection algorithm.

The flowchart of the proposed algorithm is shown in Figure 1, in which some intermediate processing results are demonstrated for a better illustration. The main steps of the proposed algorithm are given as follows.

Step 1: coarse detection.

In the beginning, we try to get the coarse target detection result by using the traditional CFAR techniques, such as the CA-CFAR detector or the truncated statistics CFAR (TS-CFAR) detector. We demonstrate the proposed algorithm by Figure 1 to improve clarity. The SAR image adopted here for illustration consists of land, sea, and the interested ships. As can be seen from Figure 1, there are many false alarms in the land areas by adopting the CFAR detector for the image covered by complex textures (both sea and land). Since the CFAR detector only considers the intensity difference, it is hard to make a distinction between the manmade clutters and the targets.

Step 2: land-sea segmentation.

The main idea of this part is to realize accurate segmentation between the sea area and the land area. Moreover, the approach to realize the goal in this paper is to judge the statistical property of the superpixels. The processing procedures are given in detail in the following for this step.

(1) Multiscale superpixel segmentation.

Adopting different scales of superpixel segmentation means that the sizes of the superpixels are different, i.e., for a fixed image, a greater number of the total superpixels mean that the covering area of each superpixel is smaller, and a smaller number of the total superpixels imply that the covering area of each superpixel is larger. Multiscale superpixel segmentation results of the SAR image can be obtained by using the simple linear iterative clustering (SLIC) algorithm [24].

(2) Superpixel type determination.

**Figure 2:**
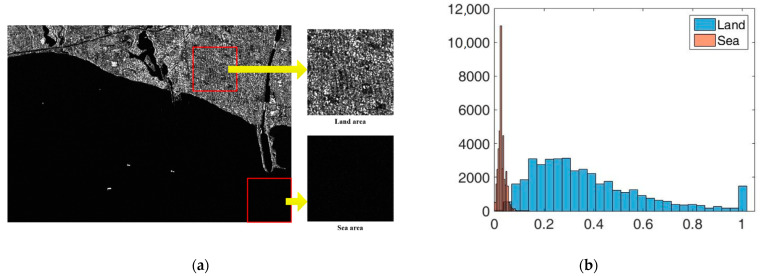
Comparisons of the land area and the sea area. (**a**) Extracted subimages of the land area and the sea area; (**b**) the histograms of the land area and the sea area.

Having the superpixel segmentation results in each scale, we will determine the types of the superpixels by using the following method. Firstly, we extract subimages from the original SAR image in both of the land areas and the sea areas, as indicated by the red squares in Figure 2a. Then, we plot the histograms of the land areas and the sea areas respectively, as shown in Figure 2b. From Figure 2b, we can see that the statistical properties of the land areas and the sea areas are totally different. In this paper, we realize land-sea segmentation of the SAR image based on the statistical property. Moreover, we choose the KL divergence [25] and the KS distance [26] to determine the type (land or sea) of a superpixel.

In the following, we give a brief description of the KL divergence and the KS distance. The KL divergence [25] can be described as

(1) $$D_{KL}\left(p||q\right)=\int p\left(m\right)\log_{2}\frac{p\left(m\right)}{q\left(m\right)}dm$$

where p and q denote the two measured probability density functions (PDFs), whereas p(m) and q(m) are the corresponding values of p and q at point m. log is the logarithmic function. Moreover, the discrete version of (1) can be expressed as

(2) $$\begin{array}{cc} D_{KL}\left(p||q\right) & =\sum p\left(m\right)\Delta m\log_{2}\left[\frac{p\left(m\right)\Delta m}{q\left(m\right)\Delta m}\right]\\ & =\sum P\left(m\right)\log_{2}\left[\frac{P\left(m\right)}{Q\left(m\right)}\right] \end{array}$$

where Δm is the increment of m, P(m) and Q(m) represent the values of the probabilities. As can be seen from (2), $D_{KL}\left(p||q\right)\neq D_{KL}\left(q||p\right)$. As a result, the KL divergence is calculated by using $D_{KL}=D_{KL}\left(p||q\right)+D_{KL}\left(q||p\right)$ in this paper. That is to say, if the measured two PDFs are the same, the value of $D_{KL}$ will be zero. In other words, the smaller the value of $D_{KL}$ is, the more similar the two measured PDFs will be. The land or sea judgment can be realized by searching the smaller KL value.

Another effective way of measuring the similarity is to evaluate the differences of the cumulative density functions (CDFs). Moreover, the KS distance is expressed as [26]

(3) $$D_{KS}\left(P||Q\right)=\underset{m}{\sup}\left|c_{P}\left(m\right)-c_{Q}\left(m\right)\right|$$

where $\underset{m}{\sup}\left(*\right)$ is the supremum of the function with respect to m. $c_{P}$ and $c_{Q}$ are the CDFs of the two measured PDFs of P and Q, respectively. From (3), we can tell that just like the KL divergence, the smaller the value of $D_{KS}\left(P||Q\right)$ is, the more similar the two measured CDFs will be. For instance, if a measured superpixel belongs to the sea, the KS distance between the CDF of the measured superpixel and the CDF of the sea area will be smaller than the result obtained by comparing it with the CDF of the land areas.

From the definitions and analyses of the KL divergence and the KS distance, we can see that KL evaluates the difference of two probabilities based on the PDFs, whereas KS evaluates the difference of two probabilities based on the CDFs. Both KL and KS can realize the land or sea judgment of a given superpixel. The type of a superpixel corresponds to smaller values for both KL and KS evaluations.

After judging the type of each superpixel in the SAR image, the land-sea segmentation results in different scales can be obtained.

Step 3: target detection in each scale.

The land-sea segmentation result in each scale (obtained in Step 2) and the coarse detection result obtained by using the CFAR detector (obtained in Step 1) are combined together to eliminate the false alarms in this step. As a result, we can obtain the detection result in each scale. Disturbances in the land areas can be suppressed by the combination.

Step 4: obtain the final detection result.

The superpixel segmentations in different scales can provide different aspects of the local features of the SAR image. Moreover, fusing the detection results under different scales together will lead to a better description and understanding of the image.

In the final step, we will fuse the detection results in different scales to improve the performance of the proposed target detection algorithm. The reason why we adopt fusion is due to the fact that some superpixels contain both land and sea, and the land-sea segmentation results are different under different superpixel scales, especially in the regions near the coastline. Fusing multiple detection results obtained under different scales can further eliminate the false alarms in the sensitive regions. In other words, the fusion can remove the disturbances derived from manmade clutters in the land area, and improve the detection performance near the coastline. The fusion can be given by

(4) $$y_{final}=y_{1}•y_{2}•\ldots •y_{N}$$

where $y_{final}$ is the final detection result, $y_{i}\left(i=1,2,\ldots ,N\right)$ is the detection result in the *i*th scale obtained in Step 3, N is the number of the scales, and • denotes the element-wise multiplication.

## 3. Experimental Results and Analysis

In this part, we tested the performance of the proposed algorithm. The data used in this paper are the public SAR Ship Detection Dataset (SSDD), which consists of the SAR images collected under different conditions (both of inshore and offshore) with all polarization modes (HH, HV, VV, and VH, here, H is short for horizontal, and V is short for vertical) by the satellites of RadarSat-2, TerraSAR-X, and Sentinel-1 [27]. The resolution of the images ranged from 1 m to 15 m, and the images have been calibrated and quantified to be 0–255. In the SSDD, the ships that covered more than 3 pixels have been annotated, which can serve as the ground truth for performance evaluation of the target detection algorithms.

We conducted the experiments in three different cases by using different CFAR detection methods to validate the robustness of the proposed algorithm on coarse detection result. Moreover, we compared the proposed algorithm with the superpixel-based CFAR detection algorithm [28] to validate the advantage of fusion. In the first case, we conducted the coarse detection by utilizing the widely used CA-CFAR detector [16]. In the second case, the TS-CFAR detector was employed [29], which was more suitable for high-target-density situations. In the third case, considering the distribution of the sea clutter, we employed the Weibull-CFAR detector [30] to achieve the coarse detection result.

**Figure 3:**
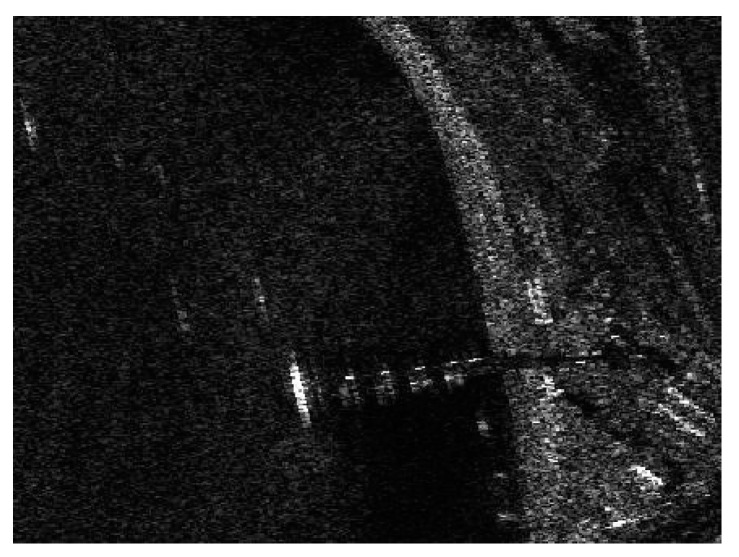
SAR image of SAR Ship Detection Dataset (SSDD).

**Figure 4:**
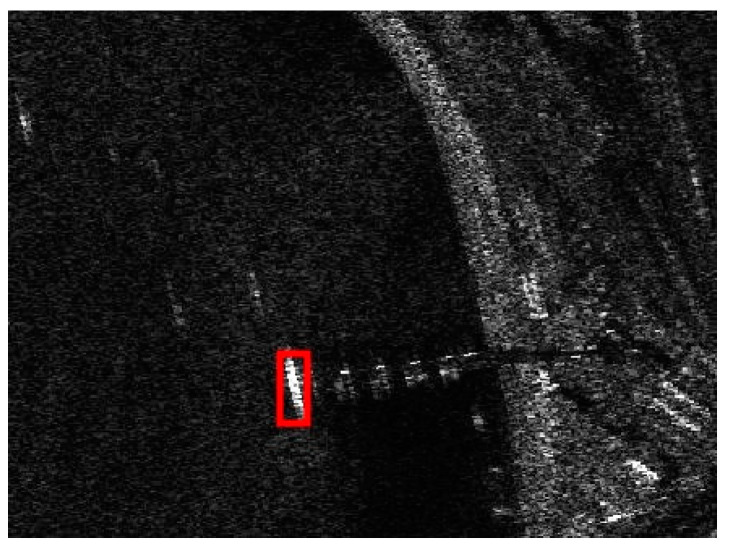
The ground truth.

**Figure 5:**
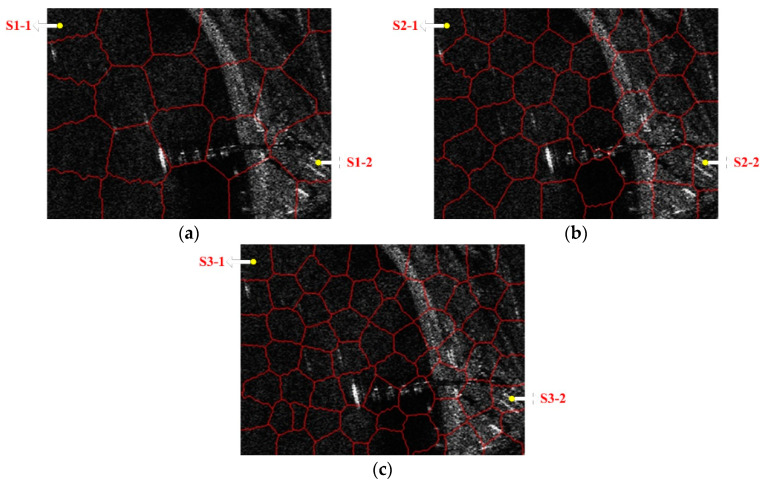
Superpixel segmentation results of the chosen SSDD image under different scales.(**a**) Scale 1; (**b**) Scale 2; (**c**) Scale 3.

**Figure 6:**
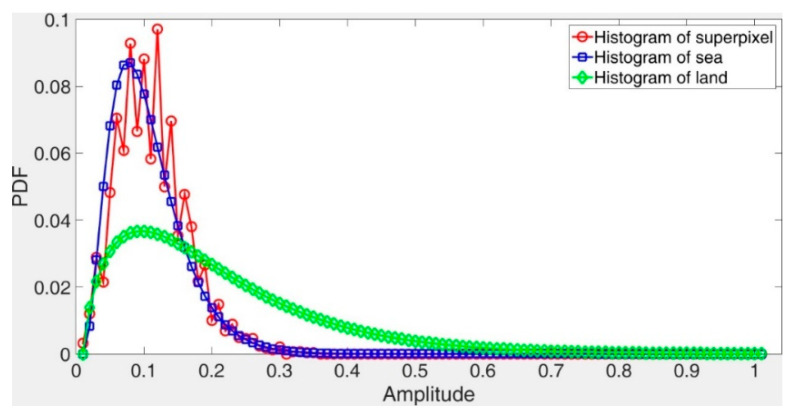
Probability density functions (PDFs) of the superpixel, the sea, and the land.

In the first case, we validated the effectiveness of realizing land-sea segmentation by using the statistical property of the superpixels in the beginning. Taking a SAR image of SSDD as an illustration, the chosen image is displayed in Figure 3, and the corresponding ground truth is given in Figure 4. The superpixel segmentation results, under three different scales, are shown in Figure 5. The number of the superpixels is set to be 50, 100, and 150 for Scale 1, Scale 2, and Scale 3, respectively. Actually, we can also change the number of the superpixels. The main principle of determining the number of the superpixels is to make sure that the target will not cover the most areas of one superpixel in case that the type of the superpixel that contains the target be judged into land. In other words, if most areas of a superpixel are covered by an interested target, the statistical property of the superpixel will be more similar to land than to sea. In other words, the type of the superpixel will be judged into land, which will result in wrong elimination of the target. The superpixels of S1-1, S2-1, and S3-1 correspond to the same sea area, and the superpixels of S1-2, S2-2, and S3-2 correspond to the same land area, as demonstrated in Figure 5. The KL and KS values of the selected superpixels are shown in Table 1. (2) Corresponds to the KL divergence, whereas (3) corresponds to the KS distance. Here, we give an example of the KL divergence. The PDFs of the superpixel S1-1, the sea, and the land are displayed in Figure 6. If we calculate the distance between the superpixel and the sea, P(m) will be assigned the values represented by the red circles shown in Figure 6, and Q(m) will be assigned the values represented by the blue rectangles shown in Figure 6. Similarly, if we need to calculate the distance between the superpixel and the land, P(m) will be assigned the values represented by the red circles shown in Figure 6, and Q(m) will be assigned the values represented by the green diamonds shown in Figure 6.

As can be seen from Table 1, the KL and KS values calculated among the histograms of S1-1, S2-1, S3-1, and the land area are greater than those of the sea area. As a result, the type of S1-1, S2-1, and S3-1 will be judged as sea. Moreover, the KL and KS values calculated between the histograms of S1-2, S2-2, S3-2, and the land area is smaller than those of the sea area. Therefore, the type of S1-2, S2-2, and S3-2 will be judged as land, accordingly.

As can be seen, all types of the superpixels are correctly judged, leading to accurate land-sea segmentations. Satisfying land-sea segmentation results can be obtained by using the statistical property of the superpixels.

**Figure 7:**
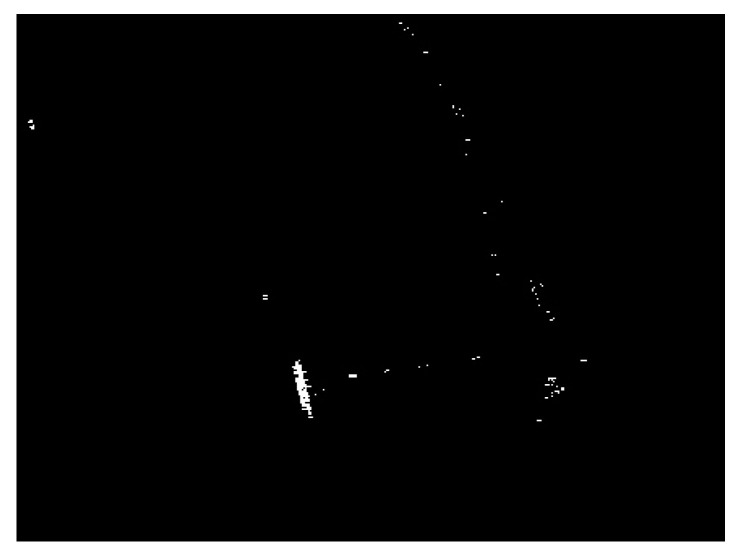
Detection result of the cell-averaging constant false alarm rate (CA-CFAR) detector for the chosen SSDD image.

The detection result of the chosen SSDD image obtained by using the CA-CFAR detector is demonstrated in Figure 7. The key parameters of the CA-CFAR detector are given as follows: the size of the guard area is set to be 80 × 80 pixels, the size of the background area is set to be 100 × 100 pixels, and the false alarm rate is set to be 0.001. From Figure 7, we can see that the interested target in the sea area can be detected accurately. However, there are still many false alarms, by only considering the intensity differences under the CA-CFAR detection. We should eliminate as many false alarms as possible.

**Figure 8:**
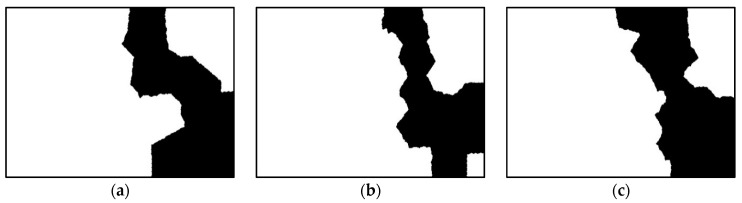
Land-sea segmentation results of the chosen SSDD image under different superpixel scales. (**a**) Scale 1; (**b**) Scale 2; (**c**) Scale 3.

**Figure 9:**
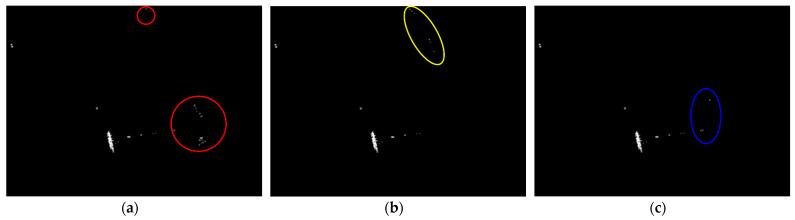
Detection results of the chosen SSDD image under different superpixel scales in the first case. (**a**) Scale 1; (**b**) Scale 2; (**c**) Scale 3.

**Figure 10:**
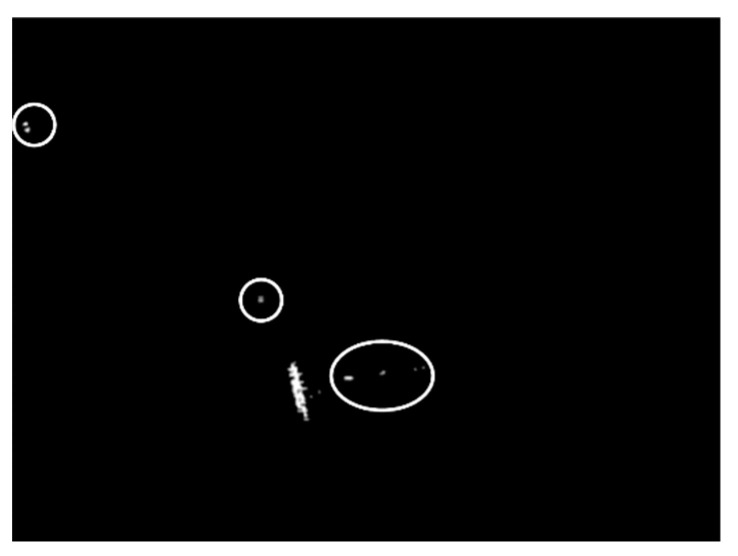
Final detection result of the chosen SSDD image in the first case.

Land-sea segmentation results under different superpixel scales are given in Figure 8. Moreover, the detection results obtained under different scales by fusing the corresponding land-sea segmentation result with the CA-CFAR detection result are displayed in Figure 9. From Figure 9, we can see that many false alarms that existed in Figure 7 have been eliminated. Inspecting Figure 9, we can further find that most of the left false alarms, after combining the land-sea segmentation with the CA-CFAR detection in different superpixel scales, lie in different locations, as shown in the red circles in Figure 9a, the yellow circles in Figure 9b, and the blue circles in Figure 9c. The reason why this happens lies in the fact that the land-sea segmentation results are different under different superpixel scales. Different scales provide different information of the local features. Single scale information is not sufficient to describe the sensitive regions. In other words, fusion of multiscale results in better descriptions of the sensitive regions. The final detection result obtained by fusing the detection results obtained in different scales together is demonstrated in Figure 10. As can be seen from the comparison between Figure 7 and Figure 10, explicit improvements can be achieved by using the proposed superpixel-based fusion detection algorithm.

**Figure 11:**
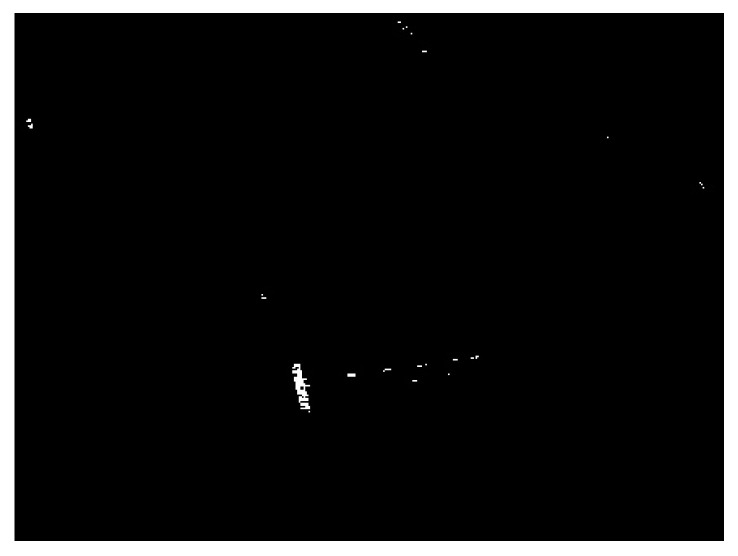
Detection result obtained by using CA-SPCFAR.

To show the effectiveness of fusion more clearly, we compare the proposed algorithm with the cell-averaging superpixel-level CFAR (CA-SPCFAR) detection algorithm presented in [28], in which the guard area and the background area are determined by using the superpixel technology. The detection result obtained by using the CA-SPCFAR is demonstrated in Figure 11. The key parameters are set as follows, the number of the superpixels is set to be 150, the compactness is set to be 40, and the false alarm rate is set to be 0.001. Comparing Figure 11 with Figure 7, we can see that the performance of the CA-SPCFAR is much better than the CA-CFAR, because superpixels can provide more precise information, with respect to the normal rectangular areas. As for the proposed algorithm, it outperforms the CA-SPCFAR, which demonstrates the effectiveness of multiscale fusion. The comparison further validates the fact that single information extraction is not sufficient for satisfying target detection results.

To show the advantage of the proposed algorithm more quantitatively, we use some scientific indicators here. We evaluate the proposed algorithm based on the detection rate $P_{d}$, the false alarm rate $P_{f}$ and the *F*_1_ score, respectively. The indicators can be expressed as [31,32]

(5) $$P_{d}=\frac{N_{\mathrm{td}}}{N_{\mathrm{ground}\_\mathrm{truth}}}$$

(6) $$P_{f}=\frac{N_{\mathrm{fd}}}{N_{\mathrm{total}\_\mathrm{detection}}}$$

(7) $$F_{1}=2\times \frac{P_{d}\times \left(1-P_{f}\right)}{P_{d}+\left(1-P_{f}\right)}$$

where $N_{\mathrm{td}}$ is the number of the correctly detected pixels in the detection result, $N_{\mathrm{ground}\_\mathrm{truth}}$ is the number of the pixels of the ground truth, $N_{\mathrm{fd}}$ is the number of the false alarms in the detection result, and $N_{\mathrm{total}\_\mathrm{detection}}$ is the number of the pixels in the detection result.

We compare these indicators obtained by using different methods; the values are obtained by using the CA-CFAR, the CA-SPCFAR, combining the CFAR detector with the land-sea segmentation under scale 1, combining the CFAR detector with the land-sea segmentation under scale 2, combining the CFAR detector with the land-sea segmentation under scale 3, and the proposed algorithm by fusing different scales, respectively. The corresponding evaluation results under different methods are given in Table 2. As can be seen, the performance of the proposed algorithm is the best. It can achieve the highest $P_{d}$ and the lowest $P_{f}$, which leads to the highest $F_{1}$. As can be seen from Table 2, the detection rate of the proposed algorithm is the same as the CA-CFAR detector, since the proposed algorithm takes the CA-CFAR detector to obtain the coarse detection result. Moreover, we can see that the fusion just eliminates the false alarms, but leaves all the true detections successfully. As for the false alarm rate, the proposed algorithm has an explicit advantage over the competitors; this is due to the fact that the main advantage of the proposed algorithm is to eliminate the false alarms obtained under different scales. Inspecting Table 2 and Figure 9, we can further tell that the false alarms are different under different scales, which further validates the significance of multiscale superpixel fusion.

**Figure 12:**
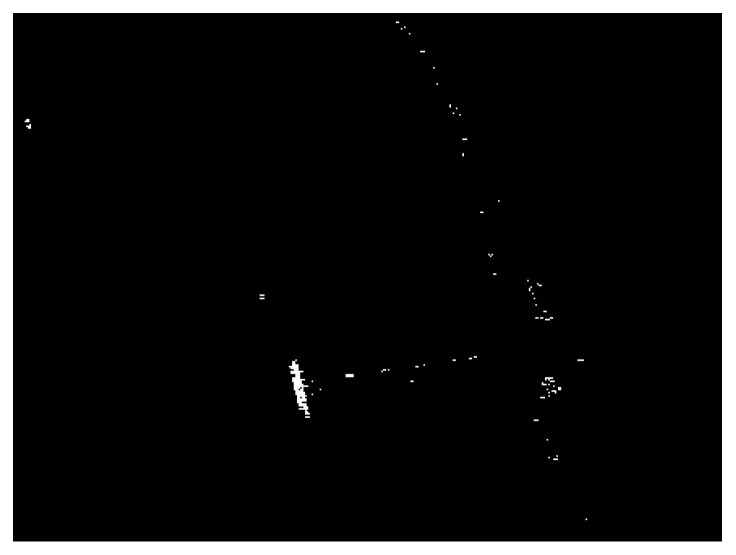
Detection result of the the truncated statistics CFAR (TS-CFAR) detector for the chosen SSDD image.

**Figure 13:**
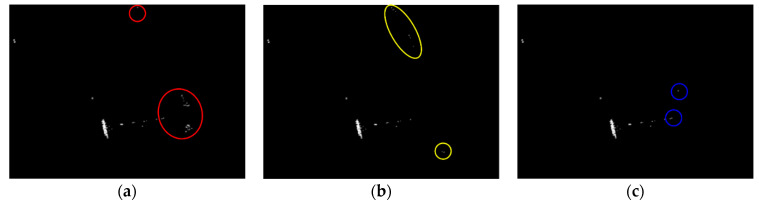
Detection results of the chosen SSDD image under different superpixel scales in the second case. (**a**) Scale 1; (**b**) Scale 2; (**c**) Scale 3.

**Figure 14:**
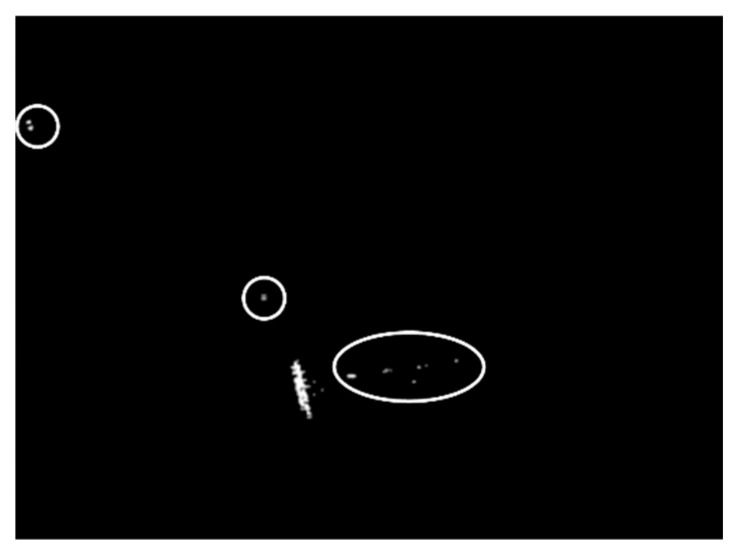
Final detection result of the chosen SSDD image in the second case.

**Figure 15:**
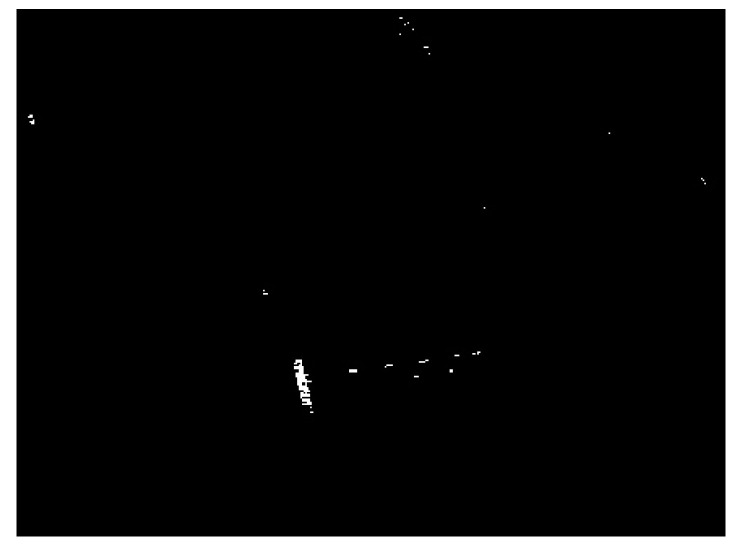
Detection result obtained by using TS-SPCFAR.

**Figure 16:**
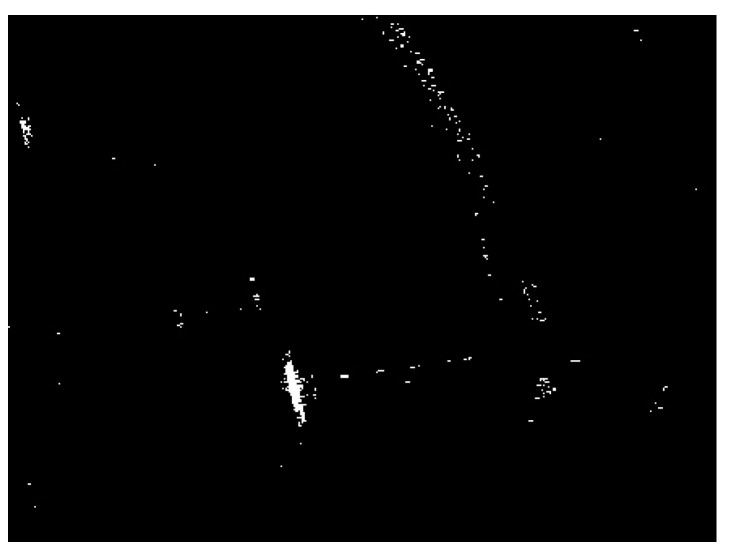
Detection result of the Weibull-CFAR detector for the chosen SSDD image.

**Figure 17:**
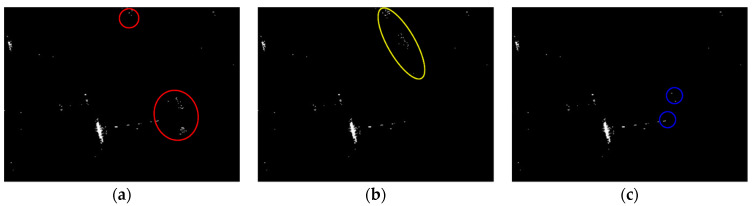
Detection results of the chosen SSDD image under different superpixel scales in the third case. (**a**) Scale 1; (**b**) Scale 2; (**c**) Scale 3.

**Figure 18:**
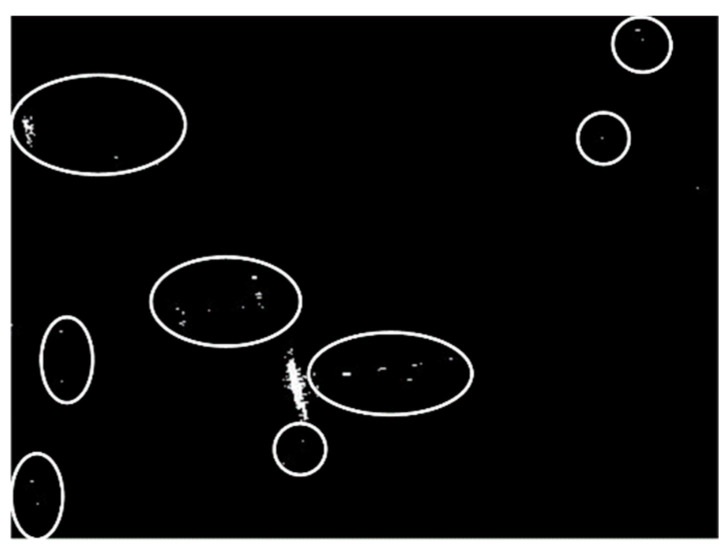
Final detection result of the chosen SSDD image in the third case.

**Figure 19:**
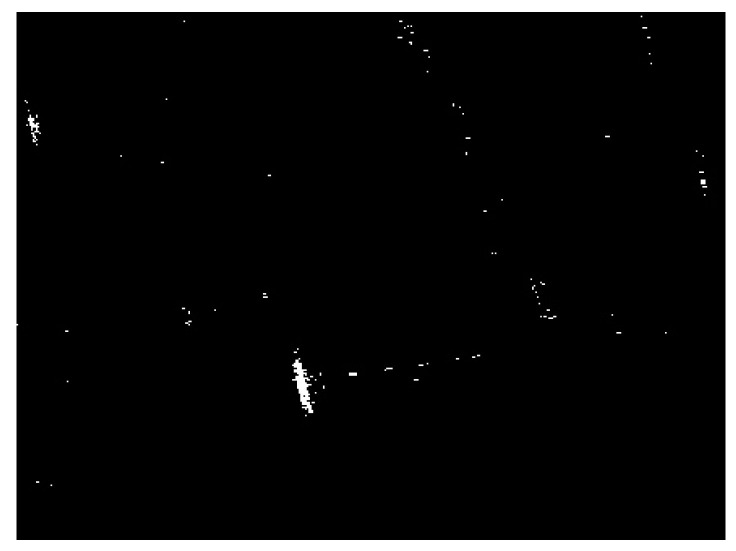
Detection result obtained by using Weibull-SPCFAR.

In the following, to test the robustness of the proposed algorithm with respect to the choice of the CFAR detectors, we conduct the proposed algorithm by using the TS-CFAR and the Weibull-CFAR to obtain the coarse detection result. The TS-CFAR fits the situation of high-target-density, whereas the Weibull distribution is more suitable for the description of sea clutters. The coarse detection result obtained by using TS-CFAR is displayed in Figure 12, and the corresponding detection results of fusing land-sea segmentation results under different superpixel scales with the coarse detection result are demonstrated in Figure 13. The final detection result is displayed in Figure 14. Similarly, we also compare the proposed algorithm with the truncated statistics superpixel-level CFAR (TS-SPCFAR) presented in [28], as illustrated in Figure 15. The corresponding results under the Weibull-CFAR are given in Figure 16, Figure 17 and Figure 18, and the result of the Weibull superpixel-level CFAR (Weibull-SPCFAR) [28] is displayed in Figure 19. From the results, we can see that the proposed algorithm has an explicit advantage. Many false alarms have been eliminated by multiscale fusion.

Experimental results of all the three experiments with different coarse detection methods demonstrate the robustness of the proposed algorithm. The proposed algorithm is not sensitive to the coarse detector, since the information of different scales is captured and utilized by fusion.

Likewise, we evaluate the detection performance of these two cases by using $P_{d}$,$P_{f}$, and $F_{1}$, respectively. The detection performance in the second case is given in Table 3, and the detection performance in the third case is demonstrated in Table 4. From the results, we can see that, just like case 1, the proposed algorithm can achieve the highest detection rates and the lowest false alarm rates. Fusing information of different scales eliminates the false alarms effectively.

However, false alarms still exist after fusing multiscale information, as shown in the white circles of Figure 10, Figure 14, and Figure 18. The phenomenon demonstrates the fact that, although fusing the multiscale superpixel contributes to disturbance suppressing, false alarms still exist. Fortunately, target detection is the first step of SAR ATR, which consists of three consecutive stages. We can still eliminate the false alarms in the discrimination stage and the recognition (classification) stage.

Finally, we give a brief discussion about the computational complexity of the proposed algorithm. Since the proposed algorithm fuses the CFAR detector and the superpixel technology together, its computational complexity depends on the computational complexity of the CFAR detector and the superpixel technology. Fortunately, the computational complexity of the CFAR detector and the superpixel technology are both low [24,33]. For instance, the computational complexity of the CA-CFAR detector is O(n) to determine the thresholds, whereas the computational complexity of SLIC is also O(n), where n is the number of pixels in the image. If we combine them together, the computational complexity of the proposed algorithm will still be O(n).

## 4. Conclusions

A target detection algorithm for complex SAR imaging scenes based on CFAR detectors and multiscale superpixel fusion is proposed in this paper. Traditional CFAR detectors are used to achieve the coarse detection result, and the land-sea segmentation, which can better describe the features and the properties of the SAR images, is conducted by using the superpixel technology. Experimental results have demonstrated that the superpixel technology can give a precise description of the sensitive complex regions, and different scales of the superpixels can provide different information. Fusing multiscale superpixel can suppress the false alarms effectively, especially for the manmade clutters near the coastline. Making use of the statistical property can provide discriminative power for land-sea segmentation. From the experimental results, we can find that owing to the effectiveness of fusion, the proposed algorithm is not sensitive to the coarse detection result—or better, different choices of the CFAR detectors will not lead to obvious differences on the final detection result.

Moreover, the number of the scales and the number of the superpixels are not fixed, and one can adjust them according to practical applications.

Note that, although the proposed algorithm can achieve satisfying target detection results for the complex imaging scenes, false alarms cannot be eliminated completely. How to reduce the stubborn false alarms still deserves further studying.

## Figures and Tables

**Table 1:** The Kullback–Leibler (KL) and Kolmogorov–Smirnov (KS) values for different superpixels under different scales.

Measures		KL		KS		Decision
Type		Land	Sea	Land	Sea	
Scale 1	S1-1	0.3512	0.0484	0.3808	0.0891	Sea
	S1-2	0.0400	0.8206	0.0417	0.3839	Land
Scale 2	S2-1	0.3534	0.1068	0.3455	0.1209	Sea
	S2-2	0.0607	1.2281	0.0602	0.4482	Land
Scale 3	S3-1	0.3270	0.0511	0.3707	0.0997	Sea
	S3-2	0.0725	1.0640	0.0401	0.3811	Land

**Table 2:** Detection performance in the first case.

Algorithms	$P_{d}\;(\%)$	$P_{f}\;(\%)$	$F_{1}$
CA-CFAR	87.28	44.07	0.6817
CA-SPCFAR	75.14	32.64	0.7104
Combining the CFAR detector with the land-sea segmentation under scale 1	87.28	34.91	0.7457
Combining the CFAR detector with the land-sea segmentation under scale 2	87.28	25.98	0.8011
Combining the CFAR detector with the land-sea segmentation under scale 3	87.28	24.88	0.8075
The proposed algorithm by fusing different scales	87.28	22.56	0.8207

**Table 3:** Detection performance in the second case.

Algorithms	$P_{d}\;(\%)$	$P_{f}\;(\%)$	$F_{1}$
TS-CFAR	87.86	49.33	0.6427
TS-SPCFAR	75.14	35.96	0.6915
Combining the CFAR detector with the land-sea segmentation under scale 1	87.86	39.44	0.7170
Combining the CFAR detector with the land-sea segmentation under scale 2	87.86	29.30	0.7835
Combining the CFAR detector with the land-sea segmentation under scale 3	87.86	27.27	0.7958
The proposed algorithm by fusing different scales	87.86	25.12	0.8085

**Table 4:** Detection performance in the third case.

Algorithms	$P_{d}\;(\%)$	$P_{f}\;(\%)$	$F_{1}$
Weibull-CFAR	97.11	70.47	0.4528
Weibull-SPCFAR	91.33	58.85	0.5673
Combining the CFAR detector with the land-sea segmentation under scale 1	97.11	60.00	0.5666
Combining the CFAR detector with the land-sea segmentation under scale 2	97.11	58.52	0.5813
Combining the CFAR detector with the land-sea segmentation under scale 3	97.11	55.08	0.6143
The proposed algorithm by fusing different scales	97.11	53.97	0.6245

## Data Availability

The data used to support the findings of this study are available from the corresponding author upon request.
